# Editorial: Added sugar consumption: economic and policy perspectives for improving public health

**DOI:** 10.3389/fnut.2024.1379946

**Published:** 2024-02-23

**Authors:** Gemma Bridge, Angela Jackson-Morris, Luana Silva Monteiro

**Affiliations:** ^1^School of Earth and Environment, University of Leeds, Leeds, United Kingdom; ^2^York Business School, York St John University, York, United Kingdom; ^3^RTI International, Durham, NC, United States; ^4^Instituto de Alimentação e Nutrição, Universidade Federal do Rio de Janeiro, Rio de Janeiro, Brazil

**Keywords:** non-communicable diseases, NCDs, added sugar, public health, economic evidence, policy

## Introduction

Since 1990, the number of global deaths attributable to non-communicable diseases (NCDs), such as cardiovascular disease, obesity, and diabetes has increased each year (1). According to the World Health Organization (WHO), as of 2023, every year over 41 million people die because of NCDs, equivalent to 74% of all global deaths (2). Many of these deaths occur before the age of 70, and 86% of these premature deaths occur in low- and middle-income countries (2). Unhealthy diets, high in fat, salt and free sugars, have been identified as a common risk factor for high burden NCDs such as diabetes (3), obesity (4), and dental caries (5). Free sugars are monosaccharides (single sugars) including glucose, fructose, and galactose, as well as disaccharides (double sugars) such as sucrose, lactose, maltose, and trehalose, added to foods and drinks by manufacturers, chefs or consumers (otherwise called added sugars), as well as sugars naturally present in honey syrups, fruit juices and fruit concentrates (6). Due to the health impacts of free sugars, nutritional guidelines recommend reducing their intake (7–9). Sugar found naturally in milk, whole fruit and vegetables does not count as free sugars.

## Key issues and questions

Addressing excessive free sugar consumption is a public health concern with societal implications. NCDs significantly increase risk of premature mortality, exert pressure on health systems, incur high healthcare expenses, and result in diminished workforce productivity and increased absenteeism due to illness (10). There is also a correspondingly high economic burden on families, communities, and national economies (11). Some governments globally have begun to implement policies to reduce free sugar consumption and mitigate adverse health and economic impacts (12). However, action lags behind the rapidly increasing NCD prevalence in most countries (13). Such policies aim to do this by restricting or eliminating exposure to major sources of free sugar, particularly among more vulnerable populations such as children and adolescents. Strategies include, for example, prohibiting vending machines in schools, taxation on high sugar foods and beverages such as candy and drinks sweetened with added sugar, encouraging or legislating to achieve product reformulation by manufacturers and caterers, regulating advertising and sponsorship in specific locations or specific population groups, and enforcing mandatory labeling (14).

Challenges to enacting and implementing effective policies to reduce free sugar consumption have been likened to parallel issues related to other major health-harming industries, such as tobacco, and gambling (15). These include libertarian concerns about limiting free trade and public choice, and concerns about possible negative impacts on economic growth (16, 17), and efforts by industry to influence policy decisions (18). Important issues and questions include identifying effective policy interventions and implementation strategies; the costs and benefits to countries of implementing policies to reduce free sugar consumption; impacts of sugar consumption and reduction policies on health equity and societal shifts to reduce sugar consumption patterns. Questions also arise regarding the role of industry in this area, for example in terms of their role in product reformulation; as well as any potential challenges that may arise in terms of policy influence.

## Research Topic

The aim of this Research Topic was to generate articles to enhance knowledge about the impacts of high free sugar consumption on health and society and provide insights into policy and program options in different contexts. The five articles in this Research Topic explore current dietary intakes, health impacts, guidelines, product labeling, and public perceptions of sugar related taxation.

## Themes covered

While it is widely accepted that reducing excess free sugar consumption is important to improving public health (19), the impact of reducing free sugar intake upon micronutrient intake is less well-understood. Basak Tukin et al. developed a food substitution model using data from 34,411 adults ≥ 20 y in the United States National Health and Nutrition Examination Survey, 2005–2018. They explored the impact on micronutrient intake when three servings of foods highest in free sugar and sodium were substituted with healthier alternatives. The authors found that replacing foods highest in added sugar led to more mean favorable micronutrient intake changes compared to replacing foods highest in sodium. However, cautioned that the composite nature of dishes containing multiple ingredients, means that food substitutions may result in both favorable and unfavorable micronutrient intake changes. Such findings highlight the need for further research to investigate how to minimize free sugar consumption while maximizing micronutrient intake.

High consumption of free sugars has been associated with some cancers (20), and evidence suggests that sugar sweetened beverage (SSB) consumption could be a modifiable factor to reduce cancer risk (21). In their opinion piece, Eshaghian et al. explore existing evidence relating SSB consumption in relation to cancer incidence and mortality. The authors present evidence supporting the positive association between SSB consumption and overall cancer risk. They hypothesize that this relationship may be due to a range of factors, including the increase in inflammatory biomarkers, serum levels of insulin-like growth factor-I (IGF-I), advanced glycation end-products, and obesity, pointing to the need for further research to better elucidate this association and its mechanisms.

Sugar-sweetened beverages have also been identified as an important contributor to free sugar intake (22). To assist consumers to make informed drink choices, in 2015 Taiwan implemented a mandatory policy requiring clear sugar content labeling on hand-shaken tea drinks. Liu et al. conducted a content analysis to investigate the information shared on the labels of 1581 hand shaken tea drinks and explored the online marketing strategies employed for such drinks in northern Taiwan. The study found that 42 out of 60 brands had clear and informative labeling, yet many half sugar (60.2%) and low-sugar (13%) drinks exceeded 25 g of sugar per cup. The authors concluded that many manufacturers failed to abide by labeling regulations potentially leading consumers to unwittingly consume excessive amounts of free sugar.

Sugar-sweetened beverage taxation has emerged as a widespread policy tool, and is one of the leading strategies advocated by the WHO (23), however such a tax has not yet been introduced in China. To explore public attitudes regarding SSB taxation, Zhang et al. conducted an online questionnaire with 881 people living in China. The authors found that among respondents, average monthly expenditure on SSBs was 44.8 ± 45.3 Yuan (RMB) (6.95 ± 7.02$USD). Over half of respondents (54.6%) stated that they would support an SSB tax and were willing to pay on average 1.19 times more for SSBs if a tax were to be introduced. Zhang et al., noted that support for such a tax was greater amongst those with greater awareness of the possible benefits of SSB taxation. This study highlights the importance of increasing awareness of the impact of SSBs on health and the potential benefits of an SSB tax to enhance the acceptance of such a policy in China.

In the final opinion piece within the current Research Topic, Yan and Louie suggest that policies and guidelines to limit free sugar intake should only focus on specific products. The authors argue that blanket guidance across all food and drink products may result in unintended adverse health consequences, such as promoting consumption of highly processed, but low sugar foods, including cakes, rather than natural, minimally processed foods such as fruit (24). The authors propose that guidelines should be targeted, and communications about free sugars and health for the general public should be clear and practical for them to be understood and implemented.

This Research Topic of articles adds to the body of knowledge relating to excess free sugar consumption, highlighting impacts on health and society, and provides further evidence relating to policy options, including SSB taxation, and mandatory labeling. The potential limitations of policies, and strategies to encourage wider adoption are also discussed. Nonetheless, there remain aspects of the topic that require further elucidation, including how to support people to make sustainable reductions in free sugar consumption, whether there are impact differentials between different parts of the population in different contexts. There are further policies, not explored in this Research Topic, that would benefit from research attention in different contexts, as well as research to indicate how public health experts can best explore and make the case for policies and programs with policymakers and the public. Finally, further work is needed to standardize the definition of added or free sugar so that studies can be more easily compared and understood.

## Author contributions

GB: Conceptualization, Writing—original draft, Writing—review & editing. AJ-M: Conceptualization, Writing—original draft, Writing—review & editing. LS: Writing—review & editing.
